# Motivations to participate in a Phase I/II HIV vaccine trial: A descriptive study from Dar es Salaam, Tanzania

**DOI:** 10.1186/s12889-016-2875-6

**Published:** 2016-02-24

**Authors:** E. A. M. Tarimo, M. Bakari, D. C. V. Kakoko, T. W. Kohi, F. Mhalu, E. Sandstrom, A. Kulane

**Affiliations:** Department of Nursing Management, Muhimbili University of Health and Allied Sciences, Dar es Salaam, Tanzania; Department of Internal Medicine, Muhimbili University of Health and Allied Sciences, Dar es Salaam, Tanzania; Department of Behavioural Sciences, Muhimbili University of Health and Allied Sciences, Dar es Salaam, Tanzania; Department of Microbiology and Immunology, Muhimbili University of Health and Allied Sciences, Dar es Salaam, Tanzania; Venhalsan, Karolinska Institutet, Sodersjukhuset, Venhalsan, Stockholm, Sweden; Karolinska Institutet, Public Health, Stockholm, Sweden

**Keywords:** Motivation, Participation, HIV vaccine trial, Tanzania

## Abstract

**Background:**

The search for an efficacious HIV vaccine is a global priority. To date only one HIV vaccine trial (RV144) has shown modest efficacy in a phase III trial. With existing different HIV-1 subtypes and frequent mutations, multiple trials are needed from different geographical sites particularly in sub-Saharan Africa where most HIV infections occur. Thus, motivations to participate in HIV vaccine trials among Tanzanians need to be assessed. This paper describes the motives of Police Officers who showed great interest to volunteer in HIVIS-03 in Dar es Salaam, Tanzania.

**Methods:**

A descriptive cross-sectional study was conducted among Police Officers who showed interest to participate in the HIVIS-03, a phase I/II HIV vaccine trial in Dar es Salaam. Prior to detailed training sessions about HIV vaccine trials, the potential participants narrated their individual motives to participate in the trial on a piece of paper. Descriptive analysis using content approach and frequency distributions were performed.

**Results:**

Of the 265 respondents, 242 (91.3 %) provided their socio-demographic characteristics as well as reasons that would make them take part in the proposed trial. Majority, (39.7 %), cited altruism as the main motive. Women were more likely to volunteer due to altruism compared to men (*P* < 0.01). Researchers’ explanations about HIV/AIDS vaccine studies motivated 15.3 %. More men (19.6 %) than women (1.7 %) were motivated to volunteer due to researchers’ explanations (*P* < 0.001). Also, compared to other groups, those unmarried and educated up to secondary level of education were motivated to volunteer due to researchers’ explanation (*P* < 0.05). Other reasons were: desire to become a role model (18.6 %); to get knowledge for educating others (14.0 %); to cooperate with researchers in developing an HIV vaccine (9.5 %); to get protection against HIV infection (7.0 %), and severity of the disease within families (6.2 %). These results were supported by testimonies from both men and women.

**Conclusions:**

Participation in an HIV vaccine trial in a Tanzanian context is likely to be influenced by altruism and comprehensive education about the trial. Gender differences, marital status and education level need to be considered to enhance participation in future HIV vaccine trials.

## Background

For more than three decades HIV/AIDS remains a public health concern. To date several HIV vaccine trials have been conducted to develop an effective preventive vaccine against HIV transmission [1]. However, only one HIV vaccine candidate (RV 144) has shown a modest efficacy in a phase III trial [2]. Due to existing viral mutations, multiple efficacy trials will be required from different parts of the world. In line with immunological studies, several studies have been conducted in various parts of the world to assess people’s willingness to participate in HIV vaccine trials [3–10]. A few of such studies have been conducted in sub-Saharan African countries [11–14]. Barriers such as fear of safety of an experimental vaccine, mistrust, and various concerns have been extensively cited among potential HIV vaccine trial participants [5, 6, 15–18]. Studies on motivations to participate in HIV vaccine trials are needed particularly in sub Saharan Africa where most HIV infections occur.

Corresponding to the global efforts, Tanzania has been conducting Phase I/II HIV vaccine trials. The first phase I/II HIV vaccine trial study (HIVIS-03) was conducted in 2007 among members of the Police force in Dar es Salaam, Tanzania. Preparatory workshops (sensitization meetings) were conducted among Police Officers of all ranks prior to the trial. Willingness of these officers to participate in HIVIS-03 was at 61 % [14]. The results from willingness study led to an exploratory study to understand why 39 % would not be interested to take part given the burden of HIV infection in a Tanzanian context. The explorative study showed that participants feared possible loss of close relationships if they enrolled in an HIV vaccine trial; negative effect of the trial on the reproductive biology; interference with pregnancy norms; being unsure about risks such as the risks of acquiring HIV infection; suffering physical harm and uncertainty of the intentions of the researchers conducting the trial. Furthermore, they feared for the outcome of a medical examination which was required in the trial, in that it could reveal unknown diseases [19]. It was also deemed necessary to carry out a study that would lead to an understanding of the motives behind a sub-group of Police Officers who showed interest to volunteer for HIVIS-03 trial after coming to attend a session providing detailed explanations about the purpose of phase I/II HIV vaccine trials. Therefore, this paper describes the motives of Police Officers who showed great interest to volunteer in HIVIS-03 trial in Dar es Salaam, Tanzania.

## Methods

### Study setting

The study was conducted in Dar es Salaam, Tanzania. This was a sub-study within a large HIV and AIDS project in Tanzania that included studies of HIV incidence, laboratory reference values as well as willingness to participate in an HIV vaccine trial leading up to phase I/II HIV Vaccine trial among Police Officers [14, 19].

### Population

The study population comprised of Police Officers from Dar es Salaam, Tanzania. Police officers were chosen for this study because most of them have attained four years of secondary education, and they were easily accessible as they come from an established organization. Deliberate efforts were made to ensure that participation was not coercive in any way.

### Study design

This was a descriptive cross-sectional study based both on quantitative and qualitative data.

### Recruitment

**Fig. 1:**
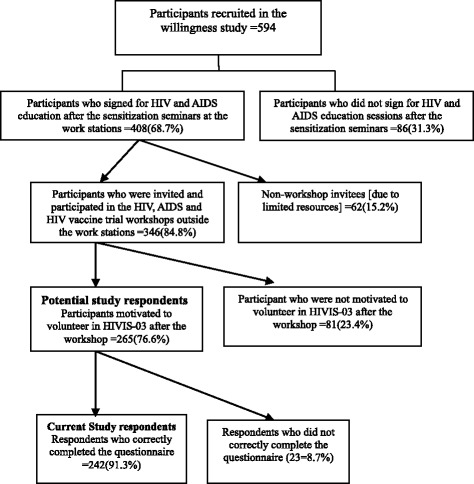
Sampling framework

Two hundred sixty five (265) participants were recruited after a series of sensitization meetings and general education sessions about HIV/AIDS and HIV vaccine trials [14, 19]. Fig. 1 narrates the sampling procedure of the participants. In brief, the trial implementers provided information about HIV and AIDS in detail. In addition, the trial implementers described all issues pertaining to HIV vaccine trials, potential benefits and risks of taking part. Examples of benefits were: complete medical check up for potential volunteers, and if they participate in the trial, they would get free medical services and referral to accredited health centres in case of illness, follow up visits and regular HIV testing. In addition, expected vaccine induced harm, such as vaccine induced seropositivity, local pain or tenderness, local redness and various systemic symptoms such malaise and headache, mild fever and nausea were described. After the workshop, several workshop attendees showed interest to volunteer for HIVIS-03, a phase I/II trial. All participants (265) who showed interest to volunteer for HIVIS-03 trial were invited for a pre-screening workshop in groups of 40 -50 individuals. This study was conducted during the HIVIS-03 pre-screening workshops (Fig. 1).

### Data collection

We collected data before the start of each pre-screening workshop. The potential volunteers were asked to fill in a questionnaire after giving consent. The questionnaire was divided into two parts. Part one consisted of socio-demographic data, and part two consisted of an open-ended question requiring the potential volunteers to narrate, in their own words, what motivated them to volunteer for an HIV vaccine trial.

The first author (EAMT) interchangeably with the second author (MB) asked the participants to anonymously narrate their views. The seating plan (chair with a writing arm) enhanced comfort ability of working individually. All questionnaires were collected and sealed in an envelope ready for data entry and analysis. Filling-in of questionnaires took 10-15 min. These data were collected between June 2006 and July 2007.

### Data analysis

#### Analysis went down in four stages

##### Stage I

The first author carefully read all the questionnaires in the original language, Kiswahili. She converted the handwritten text into an electronic format. A bilingual research scientist (ZS) translated the Kiswahili version into English. The two authors, EAMT and DCVK independently read both the Kiswahili and English texts to ensure translation consistency; minor corrections [tenses] were made. EAMT checked the completeness of personal characteristics in each questionnaire. A total of 23 respondents did not complete their socio-demographic characteristics and were excluded from analysis. Manifest content analysis approach was applied during formation of categorical statements. Categorical statements that best described what each respondent communicated were constructed to guide the next stage of analysis (Table 1).

**Table 1:** Example of socio-demographic characteristics, original text, categorical statements, and variables used for SPSS analysis

SN.	Age	Sex	Education	Marital status	Original text	Categorical Statements	Variables for SPSS
217	37	Male	Secondary School (Form IV)	Married	To know my health status; to protect my body against HIV infections when I get HIV vaccine, if I get more information about this HIV, to educate other community from not being infected against HIV as an enthusiast.	-To know my health status	kno_stat, get_prot get_edu edu_othe
						-To get protection against HIV	
						-To know more about [HIV or vaccine]	
						-To give education to others about HIV [prevention or vaccine]	

##### Stage II

To maintain integrity, EAMT and DCVK checked the categorical statements against respondents’ original text. Minor differences were discussed and agreed upon. Therefore, all categorical statement(s) that best described the respondents’ motives were moved to the next stage of analysis. A variable was formed against each categorical statement to facilitate data entry and analysis in SPSS software.

##### Stage III

The first author, transferred socio-demographic characteristics and the constructed variables (Table 1) into SPSS for windows version 20 (SPSS, Chicago, IL). The frequency distributions and proportions of all variables were tabulated. Descriptive statistics and cross tabulations to associate the socio-demographic characteristics and the selected variables were carried out. We used Pearson Chi Square Test to test significance of association.

##### Stage IV

In the final presentation, analysis shifted between the categorical statements and the original texts to select sample phrases from the respondents’ voices. To balance gender, quotes were drawn from both men and women.

### Justification of the data collection tool and analysis process

From the onset of study design, we proposed to use an open ended-question to assess individuals’ motivations to take part in the HIVIS-03. The use of elements of quantitative and qualitative analysis approaches helped to categorize and organize descriptive data into a meaningful and objective presentation.

### Ethical considerations

Ethical clearance was obtained from the Institutional Review Board at Muhimbili University of Health and Allied Sciences (MUHAS). Prospective study participants provided written consent prior to their participation. Issues of confidentiality and anonymity were strictly adhered to. Respondents were free to respond to the questionnaire and were requested not to write their names for the sake of anonymity. In spite of the presence of the study team in the venue during data collection, the respondents’ freedom was highly respected.

## Results

### Socio-demographic characteristics of the study respondents

Of the 265, a total of 242 respondents completed the questionnaire by providing their socio-demographic characteristics as well as motivations to take part in the proposed trial. The mean (SD) age was 30.7 (7.9). The majority of respondents, 76 % (184 of 242), were males. There was a gender difference with respect to age (Pearson Chi Square, *P* < 0.05); females were likely to be younger than males. All socio-demographic characteristics are summarized in Table 2. Approximately half of the males were married. Majority of females were younger, not married, and were educated up to four years of secondary education or above. It was not investigated if they had children or not.

**Table 2:** Socio-demographic characteristics of the respondents

Responses	Sex		Total, n (%)
	Men, n (%)	Women, n (%)	
Marital status			
Not married	95 (51.6)	34 (58.6)	129 (53.3)
Married	89 (48.4)	24 (41.4)	113 (46.7)
Age groups			
Up to 24	36 (19.6)	23 (39.7)	59 (24.4)*
25-34	88 (47.8)	20 (34.5)	108 (44.6)
35-44	46 (25)	12 (20.7)	58 (24.0)
45+	14 (7.6)	3 (5.2)	17 (7.0)
Education			
Primary (7 years)	54 (29.3)	18 (31)	72 (29.8)
Secondary and above (4 years)	130 (70.7)	40 (69)	170 (70.2)

Significance denoted by: * = P < 0.05

### Motivations to participate in an HIV vaccine trial

From an open-ended question, 12 categorical statements about motivations to participate in an HIV vaccine trial were recorded from the respondents. These statements were presented according to the respective socio-demographic characteristics (sex, marital status and education level) ranging from the highest to the lowest frequency in the quantitative section (Tables 3, 4, 5). Furthermore each category will be presented with supportive quotes from participants and presented in the qualitative section.

**Table 3:** Motivations for participation in an HIV vaccine trial by sex

Categorical statements	Men n(%)	Women n(%)	Total n(%)
Volunteerism to rescue lives of others [altruism]	64 (34.8)	32 (55.2)	96 (39.7)**
Desire to become a role model, to be among the enthusiasts	32 (17.4)	13 (22.4)	45 (18.6)
Researchers’ explanations about HIV/AIDS vaccine studies	36 (19.6)	1 (1.7)	37(15.3)***
To get more knowledge about HIV prevention/vaccine	27 (14.7)	10 (17.2)	37 (15.3)
To know my health status [know health status]	29 (15.8)	7 (12.1)	36 (14.9)
To get knowledge for educating others about HIV or vaccine	29 (15.8)	5 (8.6)	34 (14.0)
To cooperate with researchers in developing an HIV vaccine	21 (11.4)	2 (3.4)	23 (9.5)*
To accomplish personal motivation	14 (7.6)	5 (8.6)	19 (7.9)
To get protection against HIV infection	13 (7.1)	4 (6.9)	17 (7.0)
Magnitude of the disease [AIDS]	14 (7.6)	3 (5.2)	17 (7.0)
Severity of the disease in families – experienced death of a relative due to AIDS	13 (7.1)	2 (3.4)	15 (6.2)
Others (free treatment/insurance, test the strength of vaccine in the body)	6 (3.3)	1 (1.7)	7 (2.9)

Significance denoted by: *** = P < 0.001; ** = P < 0.01; * = P < 0.05

NB: The columns represent multiple responses, and thus cannot add up to 100 %. The same applies to the total number of respondents in each column

**Table 4:** Motivations for participation in an HIV vaccine trial by marital status

Categorical statements	Unmarried n(%)	Married n(%)	Total n(%)
Volunteerism to rescue lives of others [altruism]	52 (40.3)	44 (38.9)	96 (39.7)
Desire to become a role model, to be among the enthusiasts	24 (18.6)	21(18.6)	45 (18.6)
Researchers’ explanations about HIV/AIDS vaccine studies	25 (19.4)	12 (10.6)	37 (15.3)*
To get more knowledge about HIV prevention/vaccine	19 (14.7)	18(15.9)	37 (15.3)
To know my health status [know health status]	17 (13.2)	19(16.8)	36 (14.9)
To get knowledge for educating others about HIV/vaccine	16 (12.4)	18(15.9)	34 (14.0)
To cooperate with researchers in developing an HIV vaccine	15 (11.6)	8 (7.1)	23 (9.5)
To accomplish personal motivation	10 (7.8)	9 (8.0)	19 (7.9)
To get protection against HIV infection	7 (5.4)	10 (8.8)	17 (7.0)
Magnitude of the disease [AIDS]	7 (5.4)	10 (8.8)	17 (7.0)
Severity of the disease in families – experienced death of a relative due to AIDS	8 (6.2)	7 (6.2)	15 (6.2)
Others (free treatment/insurance, test the strength of vaccine in the body)	3 (2.4)	4 (3.6)	7 (2.9)

Significance denoted by: * = P < 0.05

NB: The columns represent multiple responses, and thus cannot add up to 100 %. The same applies to the total number of respondents in each column

**Table 5:** Motivation for participation in an HIV vaccine trial by educational level

Categorical statements	Primary n(%)	Secondary n(%)	Total n(%)
Volunteerism to rescue lives of others [altruism]	25(34.7)	71 (41.8)	96 (39.7)
Desire to become a role model, to be among the enthusiasts	24 (18.6)	21(18.6)	45 (18.6)
Researchers’ explanations about HIV/AIDS vaccine studies	4 (5.6)	33 (19.4)	37(15.3)*
To get more knowledge about HIV prevention/vaccine	10 (13.9)	27(15.9)	37 (15.3)
To know my health status [know health status]	18 (25.0)	18(10.6)	36 (14.9)*
To get knowledge for educating others about HIV/vaccine	14 (19.4)	20(11.8)	34 (14.0)
To cooperate with researchers in developing an HIV vaccine	6 (8.3)	17(10.0)	23 (9.5)
To accomplish personal motivation	8 (11.1)	11 (6.5)	19 (7.9)
To get protection against HIV infection	5 (6.9)	12 (7.1)	17 (7.0)
Magnitude of the disease [AIDS]	5 (6.9)	12 (7.1)	17 (7.0)
Severity of the disease in families– experienced death of a relative due to AIDS	3 (4.2)	12 (7.1)	15 (6.2)
Others (free treatment/insurance, test the strength of vaccine in the body)	0 (0)	7 (4.2)	7 (2.9)

Significance denoted by: * = P < 0.05

NB: The columns represent multiple responses, and thus cannot add up to 100 %. The same applies to the total number of respondents in each column

### Quantitative Section

#### Motivations by sex

There was a statistically significant difference of reasons to participate in the HIV vaccine trial between men and women. Whereas women were motivated by altruism (*P* < 0.01), men were motivated by researchers’ explanation about HIV/AIDS vaccine studies (*P* < 0.001) and intention to cooperate with researchers in developing an HIV vaccine (*P* < 0.05 (Table 3).

#### Motivations by marital status

There was a significant difference in terms of reasons to participate in the HIV vaccine trial between the married and the unmarried participants. Unlike those who were married, respondents who were unmarried were motivated to participate by the researchers’ explanations about HIV/AIDS vaccine studies (*P* < 0.05) (Table 4).

#### Motivations by education

There was a significant difference of motivations to participate in the HIV vaccine trial in relation to educational status. Compared to other levels those who attained four years of secondary education level were more likely to volunteer because of researchers’ explanations about HIV/AIDS vaccine studies (*P* < 0.05). On the contrary those who attained seven years of primary education as compared to those who attained secondary education were more likely to volunteer because of knowing their health status (*P* < 0.05) (Table 5).

### Qualitative Section

#### Volunteerism to rescue lives of others (altruism)

The majority of the respondents commonly cited altruism as a motivation to volunteer in HIVIS-03. There was a gender difference with respect to altruism where women were more likely than men to volunteer on account of altruism. They expressed their altruism as follows:

*“The thing that motivates me most is to get HIV vaccine which will be able to perish the HIV infection and save lives of many Tanzanians … especially youths and children” (26 years old, unmarried woman)*

*“I decide to volunteer in order to save lives of Tanzanians and the world at large…”(24 years old unmarried man)*

#### Desire to become a role model, to be among the enthusiasts

The desire to be a role model to others through participation in HIVIS-03 was cited by several respondents. The following statements refer to expressions of men and women:

*“I am very happy to be among the Tanzanians participating … I will be among the people who helped to stop this disease” (25 years old, unmarried man)*

*“… is to be a model or source for developing that vaccine; without me to volunteer for vaccine trial, who will do it..?” (32 years old, married woman)*

#### Researchers’ explanations about HIV/AIDS vaccine studies

Fifteen percent of the participants stated that they were motivated to volunteer in the HIVIS-03 after explanations about the nature of the HIV and/or HIV vaccine studies provided by researchers. Here more men than women were motivated by researchers’ explanations. They stated:

*“The thing that motivates me … is the education, the adequate information I received in one of the seminars…” (32 years old, unmarried man)*

*“It is after attending these seminars and understood about this HIV vaccine…” (38 years old, married woman)*

#### To get more knowledge about HIV prevention/vaccine

Some respondents were motivated to volunteer in HIVIS-03 in order to gain more knowledge about HIV/AIDS or HIV vaccine trials. They perceived the trial as an opportunity for more information:

*“I saw it is very important to join this program in order to get more understanding on how to avoid HIV infection” (30 years old, married man)*

*“Mostly is to understand, how this HIV vaccine can help to prevent this dangerous disease…” (24 years old, unmarried woman)*

#### To know my health status (Know health status)

Some of the respondents were motivated to participate based on the medical check-ups which are essential prior to enrolment into the HIV vaccine trial that will give them an opportunity to know their health status.

*“Firstly, I want to understand my health status…” (25 years old, unmarried man)*

*“The thing that motivates me until reaching the decision is to know in detail about my health status” (26 years old, unmarried woman)*

#### To get knowledge for educating others about HIV/vaccine

Others were motivated by the opinion that they could get more education about HIV and or HIV vaccine trials from the workshops and educate others:

*“… if I get more information about this HIV, I will educate other community members from not being infected with HIV…” (37 years old, married man).*

*“As a parent, I will get sufficient education through attending these seminars… to be able to educate my family against this disease [AIDS]” (42 years old, married woman).*

#### To cooperate with researchers in developing an HIV vaccine

About 11.4 % of men expressed a motivation to participate in the HIV vaccine trial as being “to cooperate with the research team in the process of developing an HIV vaccine” The corresponding proportion among the 58 women was even fewer (3.4 %).

Below are some excerpts:

*“The reason that motivates me to participate in HIV vaccine is to cooperate with the research team …the community cannot get out of this disaster without a vaccine” (28 years old, married man).*

*“The thing that motivates me to participate in HIV vaccine is in order to make a success on the whole research against HIV infection…” (21 years old, unmarried woman)*

#### To accomplish personal motivation

Those respondents motivated by their individual motives to participate in the HIV vaccine trial expressed as follows:

*“It is my personal motives” (38 years old, unmarried man)*

*“In reality, personally, what I desire most is one day a vaccine will be obtained” (24 years old, married woman)*

#### To get protection against HIV infection

Some respondents were motivated through an expectation of getting personal protection against HIV infection through the trial.

*“The thing that motivates me to participate in HIV vaccine is … I have a wife, everyone has. Between us, one can betray the other, so the vaccine will help. In my work sometimes I face environment with injured persons…this vaccine can help on such environment” (27 years old, married man)*

*“To be among the policewomen who are provided with protection against HIV …” (36 years old, married woman)*

#### Magnitude of the disease [AIDS]

Equally, a number of male and female were motivated by the overwhelming HIV epidemic, which exists both at national and global level:

*“This disease has been a threat in our nation and the world at large” (26 years old, unmarried man)*

*“The decision to participate in this HIV vaccine is that spread of HIV calamity among human beings” (27 years old, married woman)*

#### Severity of the disease in families - experienced death of a relative due to AIDS

Few respondents were motivated because of experiencing death of a close member of the family due to AIDS. They felt obliged to participate in the trial to prevent future deaths:

*“After seeing my three relatives died of AIDs and also the way I understand the importance of getting HIV vaccine in order to save lives of many people” (49 years old, married man)*

*“I have lost my relatives with AIDS; so I am ready to participate” (23 years old, unmarried woman)*

#### Others (free treatment/insurance or test the strength of the vaccine in the body)

A small number of the respondents were motivated by reasons such as obtaining free medical treatment/insurance and/or testing the strength of the vaccine in their bodies:

*“If I will be found with a problem, I will be freely treated” (28 years old, married man)*

*“In order to know if the vaccine will help or not…” (32 years old, married woman)*

Therefore, the above testimonies complement the quantitative results and increase our understanding about potential volunteers’ desire of taking part in HIVIS-03.

## Discussion

The fact that the majority of the respondents were motivated to volunteer for HIVIS-03 due to altruism is worth noting. This implies that, many respondents were concerned about the burden which HIV/AIDS has imposed upon their lives and the society at large. This corresponds to findings from previous studies where altruism has been cited as a motivation for people to participate in HIV vaccine trials [3, 20, 21]. In the present study, increasingly women were altruistic to volunteer than men implying that women were more concerned about the burden of HIV infection in their lives than men. This could be due to the fact that studies have shown that in most cases women share a greater burden of the HIV epidemic than men [22]. Although some participants stick on the phrases related to altruistic motives, they may also share experiences of losing a close relative due to HIV/AIDS. For example, death of a close relative due to AIDS can definitely motivate one to volunteer to prevent future deaths if a preventive effective vaccine could be developed.

The researchers’ explanations about HIV/AIDS, HIV vaccine development and trials appeared to motivate potential volunteers to participate in HIVIS-03 implying that vaccine trial knowledge has an impact on people’s decision to take part in vaccine trials. This motivation was higher among men suggesting that men are more likely to benefit from correct facts about HIV vaccines and trials than women. Also researchers’ explanations appear to motivate those who were unmarried and educated up to four years of secondary education or higher. These personal characteristics can influence one to make informed decision in participating in HIV vaccine trials. Previous studies documented an increasing demand for basic HIV vaccine education to address vaccine trial concepts [4, 9, 11, 12, 14, 19]. In the current study, the observed influence of researchers’ explanations and information in relation to gender, education and marital status is important.

The fact that more men than women were eager to cooperate with researchers might have been influenced by individual understanding of the purpose of phase I/II HIV vaccine trials. This kind of positive intention to help researchers in developing an HIV vaccine has been noted elsewhere [7]. On the contrary, several studies have shown that scientists conducting HIV vaccine trials are mistrusted [17, 19, 23–25], and thus research participants may display negative attitudes towards participation in HIV vaccine trials. Nevertheless, our study shows that HIV vaccine trial education may overcome this mistrust. In addition, the intention of several participants wanting to share information about HIV and vaccines with others indicates a thirst for HIV and vaccine education in the general community.

Taking into account that only a few respondents were motivated to take part in the trial by expectation of getting personal protection against HIV infection implies that potential volunteers understood that they could not expect to get protection against HIV from an experimental vaccine phase I/II trial. A previous study from the same population [14] indicated that participants were expecting to get protection against HIV by participating in an HIV vaccine trial; however, that study was done before detailed educational workshops. Similarly, elsewhere studies revealed that study participants were motivated to volunteer for HIV vaccine trials for personal protection against HIV infection [12, 16, 20, 26]. However, some of those studies were conducted before HIV vaccine trial education. It is also worth noting that increasingly the demand to know more about HIV prevention and vaccines indicate the need of provision of education specific for HIV and HIV vaccines prior to implementation of HIV vaccine trials.

### Methodological considerations

The present study was conducted in a non-random sample, which makes generalization of the results difficult. We avoided a standardized questionnaire so as not to pre-empt the individuals’ motives. Therefore, we used a questionnaire with open-ended questions to examine the diversity of motivations for participation in an HIV vaccine trial among police officers. This approach demands high level of flexibility in data analysis than would be the case if closed ended questions were used. To avoid discrepancies in interpreting the results, more than one researcher analysed the data. Negotiation for the right meaning of the respondents’ expressions was done to agree on correct categorical statement(s) to use. The prior relationship between the researchers and the respondents could influence the results. To decrease this social desirability, anonymity was emphasised during data collection. On the contrary, the incomplete socio-demographic information of the 23 respondents might have been influenced by adherence to anonymity. Nevertheless most of the respondents completed the required socio-demographic information.

## Conclusions

The results show that volunteers in HIV vaccine trials are likely to be motivated by multiple reasons. Altruism as cited by the majority may be the main motivation in an African setting where the burden of HIV is deeply experienced. Educational sessions prior to HIV vaccine trials are revealed to be crucial to increase understanding about the conduct of these trials especially in settings where HIV vaccine trials are perceived as new concepts. Therefore, participation in an HIV vaccine trial in a Tanzanian context is more likely to be influenced by altruism and comprehensive education about the trial. In addition, to enhance recruitment in future trials, gender differences, marital status and education level should be taken into consideration. Unrealistic motivation such as getting protection from an experimental vaccine albeit mentioned by few respondents should be taken into consideration during recruitment processes. Thus, the knowledge gained from this study can better inform trial implementers to consider context specific motivations during recruitment of volunteers into HIV vaccine trials.
